# Solution structure of the major fish allergen parvalbumin Sco j 1 derived from the Pacific mackerel

**DOI:** 10.1038/s41598-017-17281-6

**Published:** 2017-12-07

**Authors:** Hiroyuki Kumeta, Haruka Nakayama, Kenji Ogura

**Affiliations:** 1 Global Station for Soft Matter, Global Institution for Collaborative Research and Education, Hokkaido University, Kita 21 Nishi 11, Kita, Sapporo, 0110021 Japan; 2 Laboratory of Biomolecular Functionalities, Department of Food Science, Faculty of Bioresources and Environmental Sciences, Ishikawa Prefectural University, 1-308 Suematsu, Nonoichi, Ishikawa, 9218836 Japan

## Abstract

Although fish is an important part of the human diet, it is also a common source of food allergy. The major allergen in fish is parvalbumin, a well-conserved Ca^2+^-binding protein found in the white muscle of many fish species. Here, we studied the solution structure of the parvalbumin Sco j 1, derived from the Pacific mackerel, using nuclear magnetic resonance spectroscopy. We mapped the IgE-binding epitope proposed in a recent study onto the present structure. Interestingly, three of four residues, which were elucidated as key residues of the IgE-binding epitope, were exposed to solvent, whereas one residue faced the inside of the molecule. We expect that this solution structure can be used in future studies attempting to analyze the various IgE-binding modes of these allergens.

## Introduction

Fish is a valuable dietary source of proteins, amino acids, fatty acids, lipids, and vitamins, and it plays an important role in not only human food and health but also food culture. However, it is also among the most common sources of food allergy. Fish allergy, caused by a pathophysiological IgE-mediated immune response to specific fish proteins, can present clinically as urticaria, asthma, diarrhea, allergic contact dermatitis, and angioedema. Fish hypersensitivity is commonly encountered in coastal areas such as many European countries and Japan^1^. The allergen sources of fish are mainly derived from meat, eggs, roe, caviar, isinglass, and gelatin^2^.

The major allergen in fish is parvalbumin, a 12-kDa sarcoplasmic protein in the white muscle of many fish species containing 108–109 amino acid residues that participates in muscle relaxation^3^. It is well conserved in the amino acid sequence, highly water soluble, and resistant to heat, chemical denaturation, and enzymatic digestion^3^. The first studies to demonstrate parvalbumin identified it in Baltic cod (*Gadus callarias*) as the allergen Gad c 1^4,5^. However, subsequent studies have identified that the major food allergen is parvalbumin derived from Atlantic cod^6^ (*Gadus morhua*; Gad m 1), carp^7^ (*Cyprinus carpio*; Cyp c 1), Atlantic salmon^8^ (*Salmo salar*; Sal s 1), and Pacific mackerel^9^ (*Scomber japonicas*; Sco j 1). Previous studies have also established IgE cross-reactivity among different fish species^10^. Fish parvalbumin has several isoforms. For example, three isoforms in Atlantic herring (Clu h 1), two isoforms in carp (Cyp c 1), and four isoforms in Atlantic cod (Gad m 1) have been identified. Each parvalbumin isoform has allergenicity. Interestingly, Pacific mackerel has a single parvalbumin isoform^11^. The cross-reactivity of parvalbumin from cod, salmon, and mackerel has been studied in clinical trials^12^. According to these studies, mackerel allergy patients show allergenic reactions to not only mackerel but also other fish species. Thus, for Sco j 1 seems to have common IgE epitopes with other fish parvalbumin isoforms.

The molecular structure of parvalbumin has been well characterized by X-ray crystallography and NMR spectroscopy. Parvalbumin contains three EF-hand motifs with two Ca^2+^-binding CD sites located on the central region and EF site at the C-terminus. The N-terminal AB site is a non-functional domain. Upon Ca^2+^ binding and release, the parvalbumin structure changes in a global-folding manner; hence, parvalbumin has a function in calcium buffering and is probably involved in muscle relaxation^13^. An NMR structural and dynamics study of Gad m 1 revealed that its structure and the accessibility of putative IgE epitopes are similar to those of other parvalbumin types^14^. In a recent study, an interaction between Gad m 1 and the scFv fragment of the antibody was established using a ^1^H-^15^N HSQC chemical shift perturbation method^15^.

Hamada *et al*. purified Sco j 1 from fish meat, tested its reactivity with IgE, using ELISA, and cloned its nucleotide sequence from the cDNA library^9^. Furthermore, Yoshida *et al*. elucidated that the region containing the 21–40 amino acid residues of Sco j 1 is a major IgE epitope using chemically synthesized oligopeptides. These studies hypothesized that IgE can recognize the linear (i.e., sequential) epitope of Sco j 1^16^. Kubota *et al*. reported that heat treatment of Sco j 1 at 140 °C completely destroys its IgE reactivity^17^. In contrast, Kobayashi *et al*. showed that Ca^2+^ binding of Sco j 1 maintains its IgE reactivity by using assays in the presence or absence of Ca^2+^ chelating agents^18^. These studies showed that IgE recognizes the conformational (i.e., structural) epitope of Sco j 1. Given that both linear and conformational epitopes were found, the molecular basis underlying Sco j 1 reactivity to IgE is highly complicated and difficult to interpret. Thus, to analyze the details of recognition between Sco j 1 and IgE, further investigations are needed based on the precise three-dimensional structure of Sco j 1. However, the three-dimensional structure of Sco j 1 has not yet been reported; it has only been modeled using a computational method. This study aimed to determine the three-dimensional structure of Sco j 1 at atomic-scale resolution using NMR spectroscopy.

## Results and Discussion

### Solution structure of Pacific mackerel parvalbumin Sco j 1

**Figure 1:**
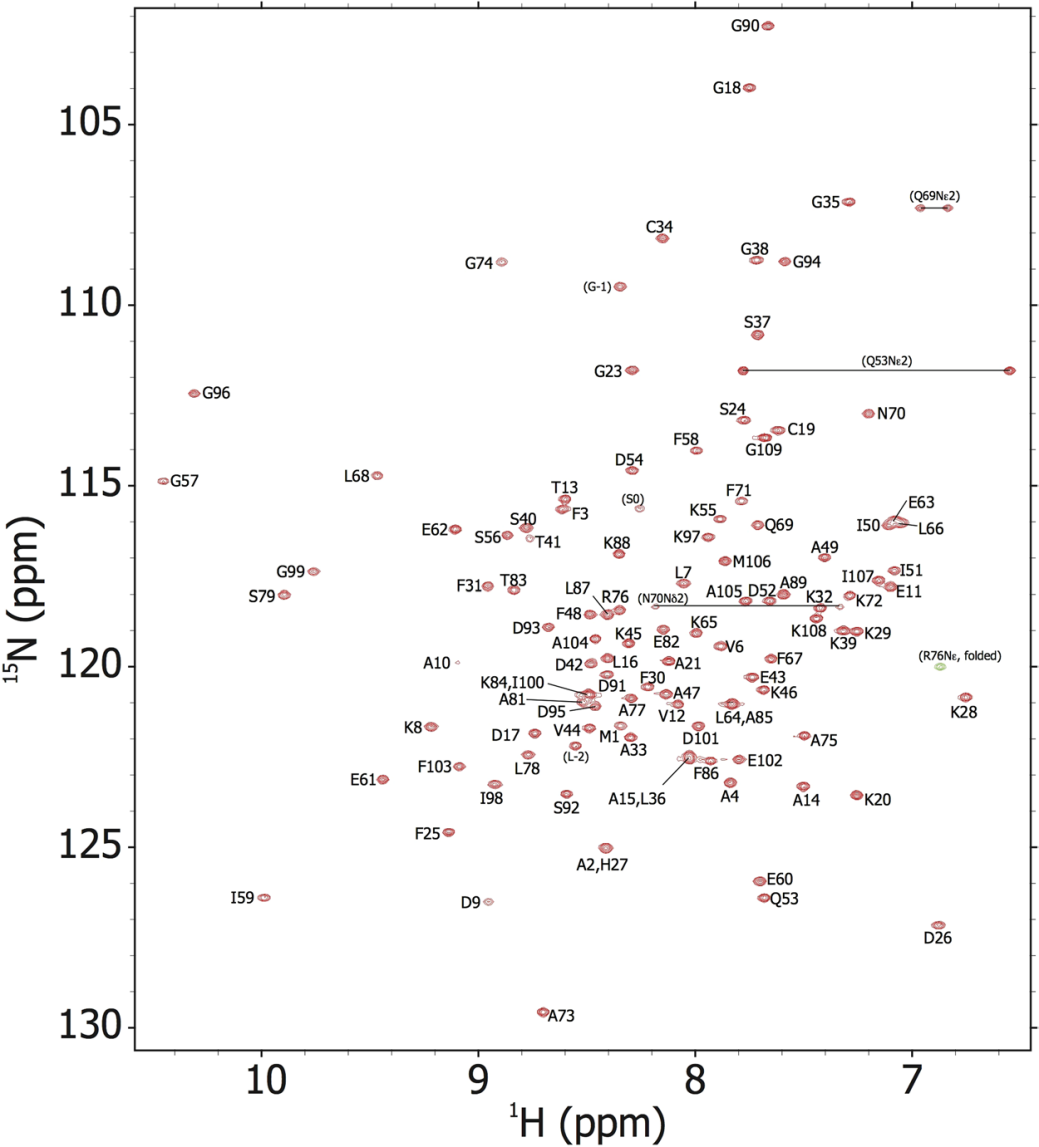
[^1^H-^15^N] HSQC spectrum of the Pacific mackerel parvalbumin Sco j 1, with resonance assignments.

**Figure 2:**
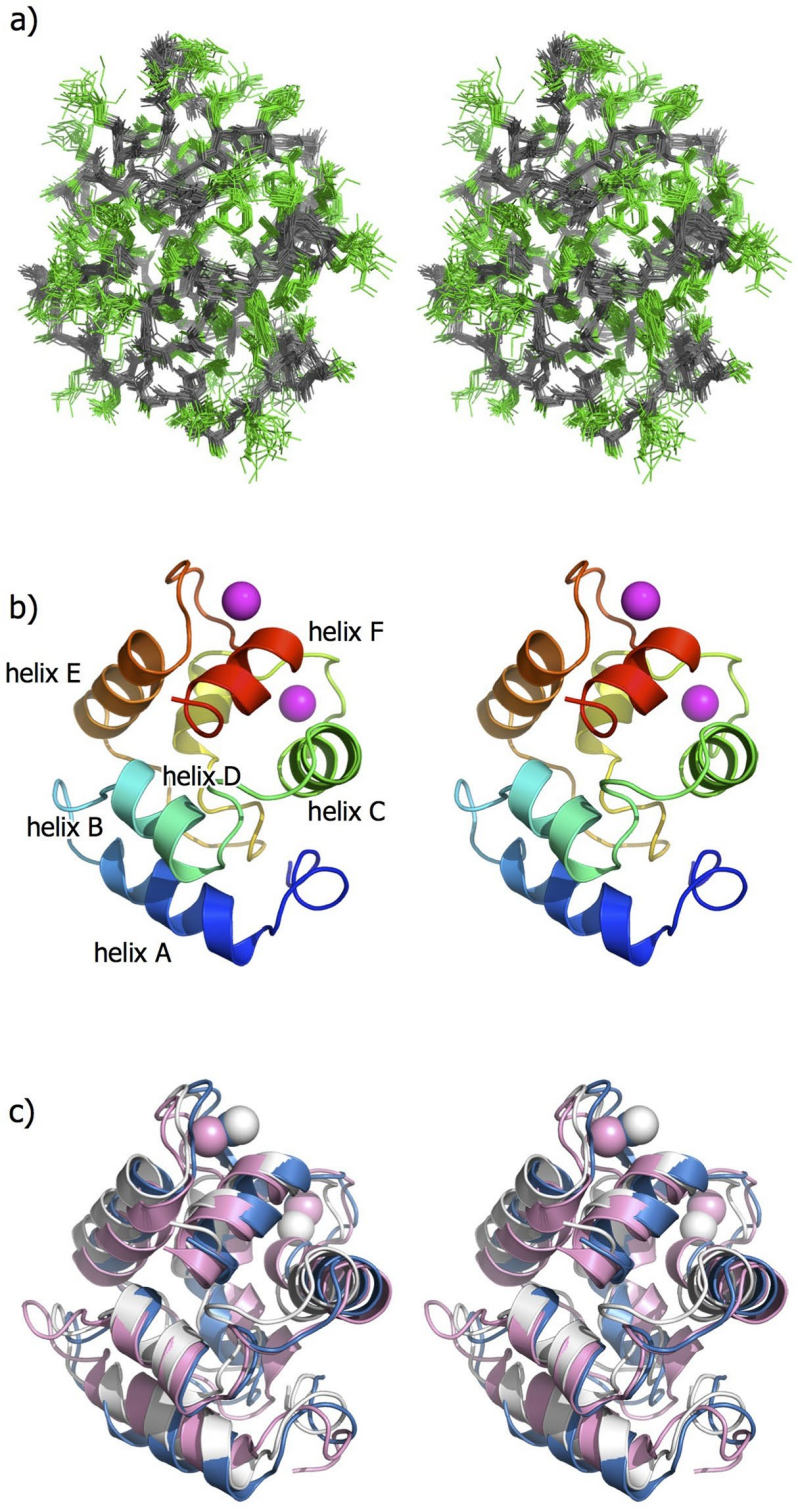
NMR structures of the Pacific mackerel parvalbumin Sco j 1 in stereo. (**a**) Overlay of the ensemble of 20 final energy-minimized CYANA structures. The main and side chains are colored in gray and green, respectively. (**b**) Ribbon diagrams of the lowest energy structure. Two Ca^2+^ ions are shown as magenta balls. (**c**) Superposition of fish allergenic parvalbumin structures of Sco j 1 (white), Cyp c 1 (cyan), and Gad m 1 (pink). All the figures were drawn using PyMOL (http://www.pymol.org/).

All of the backbone amide resonances of Sco j 1 were assigned except for Asp80 (Fig. 1). Nearly complete side-chain assignments were also accomplished. The solution structure of Sco j 1 was calculated based on 2067 inter-proton distances and 173 dihedral angle restraints. Figure 2a shows the ensemble of the 20 superimposed lowest energy structures. For residues 2–109, the mean pairwise root mean square deviations were 0.44 Å for the backbone heavy atoms and 0.80 Å for the all heavy atoms, indicating that the solution structure of Sco j 1 was well defined. Statistics of the calculated structures are listed in Table 1, showing that they satisfy the NMR-derived restraints.

**Table 1:** CYANA structure calculation statistics.

	CYANA noeassign	manual restraints
Upper distance limits		
Total	2067	889
Short-range (|i − j| ≥ 1)	1063	0
Medium-range (1 < |i − j| < 5)	411	374
Long-range (|i − j| ≥ 5)	593	515
Dihedral angle limits		
Total	173	
phi	87	
psi	86	
Target function value (Å)	0.10	
Violations		
Distance >2 Å	0	0
>1Å	1	0
Angle >1°	0	
Root mean square deviation (RMSD; Å)		
Backbone atoms	0.44	
Heavy atoms	0.80	
Ramachandran plot		
most favored region	81.3%	
additionally allowed region	18.6%	
generously allowed region	0.1%	
disallowed region	0.0%	

Figure 2b shows a ribbon representation of the lowest energy structure of Sco j 1. Sco j 1 formed a single, compact domain consisting of six α-helices with residues 9–19 (helix-A), 27–34 (helix-B), 41–51 (helix-C), 61–66 (helix-D), 80–90 (helix-E), and 100–106 (helix-F). These secondary structural elements are in good agreement with those of the other parvalbumin structures^14,19^. Helix pairs AB, CD, and EF form three EF-hand motifs. The loop regions flanked by the helix pairs CD and EF are involved in calcium binding, whereas the N-terminal AB site is a non-functional EF-hand motif.

### Comparison with other parvalbumin structures

For structural comparison, the solution structures of Sco j 1 and other parvalbumin isoforms from Atlantic cod^14^ (Gad m 1; PDB ID: 2MBX) and carp^19^ (Cyp c 1; PDB ID: 1CDP) were inputted into the DALI server (http://ekhidna.biocenter.helsinki.fi/dali_server/). The identity of amino acid sequences among fish parvalbumin is relatively high at 67–85%, suggesting that their three-dimensional structures are also similar. Sco j 1 showed high structural similarity with both Gad m 1 and Cyp c 1 (Fig. 2c and Table 2). In particular, the Z-score of 18.8 between Sco j 1 and Cyp c 1 shows that they have similar global folding structures. However, the IgE-binding conformational epitopes of parvalbumin differ with regard to not only global folding but also the electrostatic distribution, solvent accessibility, and dynamic properties of each residue. Therefore, determining the three-dimensional structure at atomic resolution is important for further investigation of allergen proteins.

**Table 2:** Structural similarity of Sco j 1 to other parvalbumins.

Allergen (PDB ID)	Z-score	RMSD (Å)	Identity (%)
Cyp c 1 (1CDP)	18.8	1.3	73.6
Gad m 1 (2MBP)	13.5	2.3	73.6

### Epitope mapping

**Figure 3:**
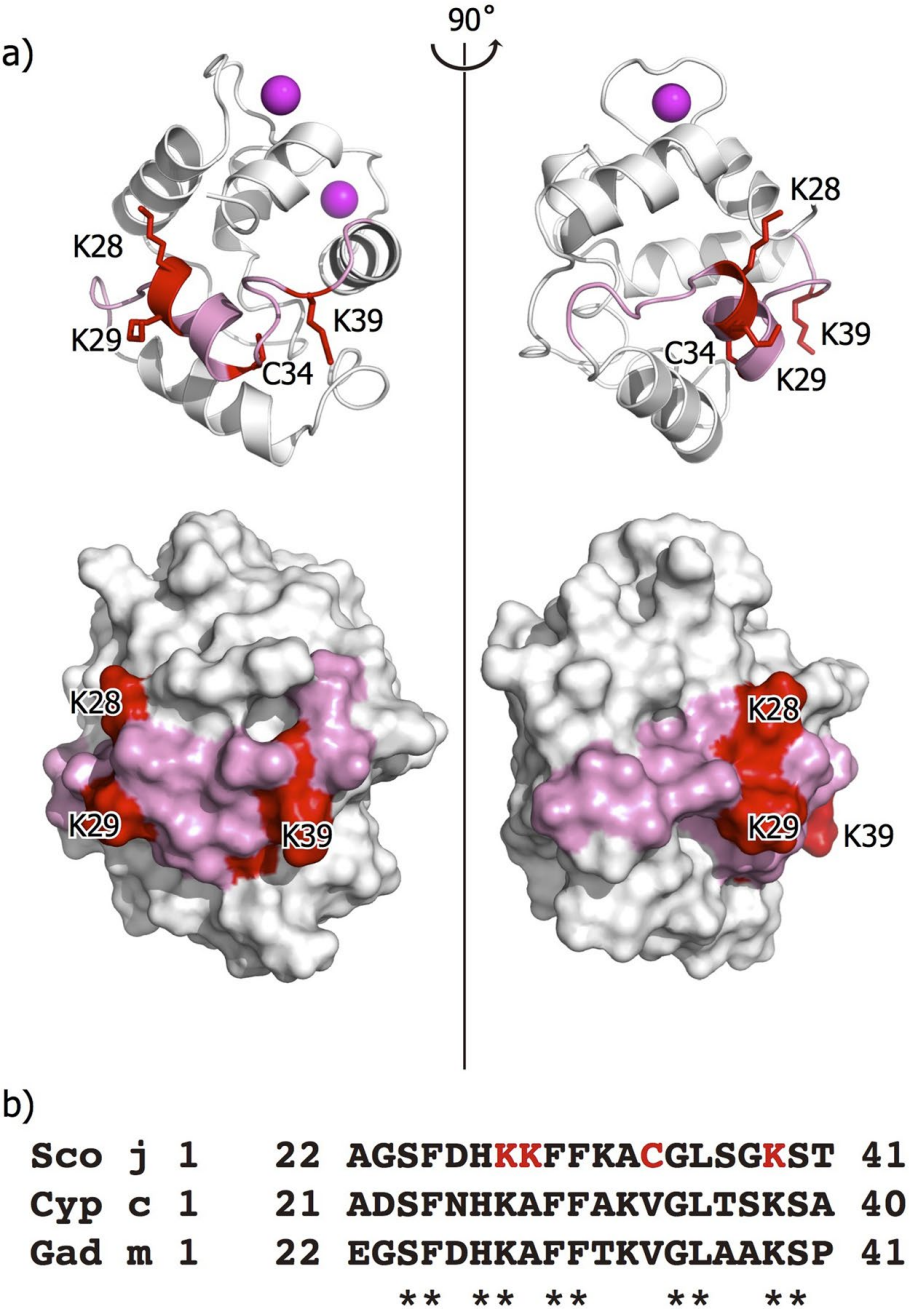
Epitope region of Sco j 1 structure. (**a**) Ribbon (upper) and surface (lower) diagram of the Sco j 1 structure. Epitope region is colored in red (Lys28, Lys29, Cys34, and Lys39) and pink (Ala22 to Thr41), respectively. (**b**) Sequence alignment of epitope region (Ala22 to Thr41) of parvalbumins Sco j 1, Cyp c 1 and Gad m 1. Lys28, Lys29, Cys34, and Lys39 of Sco j 1 are colored red. Fully conserved residues are shown by asterisk.

IgE-binding epitopes of parvalbumin have been suggested to be located in various regions. Using synthesized peptide fragments, it was previously found that the region from Ala22 to Thr41 contains the IgE-binding epitope in Sco j 1^16^. As shown in Fig. 3a, this region is located on helix-B and its surrounding region. Given that the peptide-based approach was used, this region is most likely a linear-type epitope. For Cyp c 1, the IgE-binding epitopes fall within some areas of the following residues (23, 25–29), (33–37), (77–79), and (87, 89–92, 94)^20^. The first two areas approximately overlap with the epitope of Sco j 1. Given that these epitopes were identified using the phage display technique, they are thought to be conformational epitopes. Surprisingly, for Gad m 1, the epitope is found at the C-terminal region of residues 102–109, which is quite different from the location for Sco j 1^6^.

In Fig. 3a, the sidechain atoms of Lys28, Lys29, Cys34, and Lys39 are represented by stick models. Yoshida *et al*. have shown that substitution of each residue into Ala reduces the reactivity by more than 50% in the ELISA experiments. Thus, these residues are elucidated to the key residues in the IgE-binding epitope. Whereas Lys28, Lys29, and Lys39, which are hydrophilic amino acids, are exposed to solvent, the sidechain of Cys34 faces the inside of the molecule. Thus, Cys34 is exposed to IgE binding after the molecule is decomposed in human digestive organs. Lys28 and Lys39 are fully conserved residues in Sco j1, Cyp c 1, and Gad m 1. Therefore, these residues are elucidated to play an essential role in IgE binding and crossreactivity.

As mentioned above, IgE recognizes Sco j 1 by its specific peptide region that acts as the sequential epitope. However, some evidence suggests that the IgE reactivity of Sco j 1 is maintained by its three-dimensional structure. First, substitutions of Asp51 and/or Asp90, i.e., residues consisting of Ca^2+^-binding CD and EF sites, respectively, to alanine significantly reduced IgE reactivity^21^. Similarly, the addition of ethylene glycol tetraacetic acid (EGTA) also reduces IgE reactivity^18^. These studies indicate that depletion of the Ca^2+^ binding ability results in a conformational change of parvalbumin; thus, IgE must recognize Sco j 1 by some type of conformational epitope. Second, it was previously reported that the IgE reactivity of muscle extracts from Pacific mackerel gradually decreased as the temperature was increased to 120 °C and was completely lost at 140 °C^17^. This also indicates that IgE recognizes the three-dimensional structure of parvalbumin.

In conclusion, this study investigated the solution structure of Sco j 1, a Pacific mackerel parvalbumin allergen, which was found to be similar to that of Gad m1 and Cyp c 1 derived from other fish species. The IgE-binding epitopes of Sco j 1 are located on one side of the molecular surface. Further investigation into the interaction mechanism between IgE and IgE-binding epitopes for both sequential and conformational recognition modes will be addressed using purified monoclonal antibodies for Sco j 1.

## Methods

### Protein expression and purification

The amino acid sequence of Sco j 1 was retrieved from the UniProt database (accession code P59747). The DNA encoding full-length Sco j 1 (1–109), optimized in the codon usage for *Escherichia coli*, was cloned into a pGEX-6P-1 expression vector. The actual synthesis and purification of the vector was outsourced (GenScript, Tokyo, Japan). The expression vector was transformed into BL21(DE3) competent cells. The cells were grown in 1 L MP media containing 2 g of ^15^NH_4_Cl (Shoko Science, Tokyo, Japan) and 4 g of ^13^C-labeled glucose (CIL, USA) at 37 °C. Protein expression was induced at OD_600_ = 0.7 by adding isopropyl β-D-1-thiogalactopyranoside to a final concentration of 0.5 mM. The induced cells were cultured at 37 °C for 3 h. After harvest, disruption, and centrifugation, the expressed protein was purified using a glutathione Sepharose 4B column (GE Healthcare Life Science, USA) in a bed volume of 5 mL and eluted with 10 mM reduced glutathione-containing buffer. The GST tag was excised from Sco j 1 by incubation with HRV3C protease for 12 h at 4 °C. The 10 mL of isolated Sco j 1 was purified using an ÄKTA Purifier and a HiLoad 26/600 Superdex 75 gel-filtration column (GE Healthcare Life Science, USA). The protein was concentrated to 0.5 mM using Vivaspin 15 R (Sartorius, Germany) in NMR buffer containing 7% D_2_O, 20 mM MES, 150 mM NaCl, and 10 mM CaCl_2_ at pH 6.8.

### NMR data collection, assignments, and structure calculation

NMR spectra were obtained at 25 °C using the Avance III HD 800 MHz (Bruker BioSpin, USA) and Unity INOVA 600 and 500 MHz (Agilent, USA) spectrometers. Two- and three-dimensional NMR spectra were processed using NMRPipe^22^, and the data analysis was performed using the Sparky program. ^1^H-, ^13^C-, and ^15^N-resonances were assigned using the following set of spectra: [^1^H-^15^N] HSQC, [^1^H-^13^C] HSQC, HNCO, HN(CO)CA, HNCA, CBCA(CO)NH, HNCACB, HN(CA)HA, C(CO)NH, HCCH-TOCSY, HbCbCgCdHd, and HbCbCgCdCeHe. All chemical shift values were referenced to DSS and the following frequency ratios: (^15^N/^1^H) = 0.101329118, (^13^C/^1^H) = 0.251449519^23^. Inter-proton distance restraints for structural calculation were obtained from ^13^C-edited NOESY-HSQC and ^15^N-edited NOESY-HSQC spectra, using 75 msec of mixing time. The restraints for backbone phi and psi torsion angles were predicted from chemical shifts of backbone atoms, using the TALOS + program^24^. The structure was calculated using the CYANA 2.1 software package^25^. For calculation with calcium ions we used the canonical EF-hand Ca^2+^ -binding topology. Sco j 1 has two typical EF-hands regions: D^52^QDKSGFIEEEE^63^ and D^91^SDGDGKIGIDE^102^. Two calcium ions were coordinated one by one at each binding site. The upper limit distance from the atomic group involved in coordination was 2.8 Å.

NOE distance constraints were automatically assigned and calculated using seven cycles under the “noeassign” macro of CYANA. To correct for the automatic NOE signal assignment, we used additional distance restraints derived from manual NOE assignments. Based on a model structure of parvalbumin, constructed using MODELLAR^26^ from a carp parvalbumin structure (PDB ID: 1CDP), all NOE signals were manually assigned. Among these assigned signals, we extracted the restraints between the medium- and long-range residues, and used a pseudo-atom for all optical isomeric protons. Consequently, we used 889 distance restraints with a fixed upper limit of 6 Å. At each “noeassign” cycle, 100 structures were calculated using 30,000 steps of simulated annealing, and a final ensemble of 20 structures was selected based on the CYANA target function values.

### Data availability

#### BMRB accession number

The resonance assignments have been deposited to BMRB (code: 36086).

#### PDB accession number

The atomic coordinates have been deposited to PDB (code: 5XND).
